# Single Low-Dose Ionizing Radiation Transiently Enhances Rat RIN-m5F Cell Function via the ROS/p38 MAPK Pathway Without Inducing Cell Damage

**DOI:** 10.3390/antiox14020120

**Published:** 2025-01-21

**Authors:** Jitai Zhang, Kaicen Dai, Ruike An, Chengying Wang, Xuanting Zhou, Zhujun Tian, Zhonglu Liao

**Affiliations:** 1 School of Basic Medical Science, Wenzhou Medical University, Wenzhou 325035, China; jtaizhang@wmu.edu.cn (J.Z.); 13616573805@163.com (K.D.); ark17835207163@wmu.edu.cn (R.A.); 2 School of Public Health and Management, Wenzhou Medical University, Wenzhou 325035, China; wangcy_lw@163.com (C.W.); zhouxuantingee@163.com (X.Z.)

**Keywords:** low-dose ionizing radiation, pancreatic β-cells, insulin synthesis, reactive oxygen species, p38 MAPK pathway

## Abstract

High doses of ionizing radiation (HDIR) are known to induce cellular damage, whereas low-dose ionizing radiation (LDIR) may trigger protective biological responses. Recent studies have explored the potential benefits of LDIR in treating diabetes and its complications. However, the direct effects of LDIR on pancreatic β-cells and the underlying mechanisms remain to be elucidated. This study aimed to evaluate the effects of LDIR on pancreatic β-cell functionality and elucidate the underlying molecular mechanisms involved. Rat RIN-m5F cells were exposed to LDIR (25 mGy) or HDIR (2.5 Gy) to examine changes in insulin mRNA expression, secretion, DNA damage, and apoptosis. The roles of reactive oxygen species (ROS) and the p38 mitogen-activated protein kinase (MAPK) pathway were assessed via the use of antioxidants and pathway inhibitors. The findings indicated that LDIR transiently increased both insulin synthesis and secretion without inducing apoptosis or affecting cell proliferation. In contrast, HDIR induced a significant increase in apoptosis and a marked inhibition of proliferation. LDIR was observed to temporarily increase ROS production, activating the p38 MAPK pathway and facilitating insulin synthesis via the upregulation of PDX-1. Notably, LDIR did not induce DNA double-strand breaks or activate the ATM-dependent DNA repair pathways, unlike HDIR, which induced apoptosis through overactivation of the ROS/p38 MAPK pathway. In conclusion, LDIR enhanced pancreatic β-cell functionality via ROS-mediated activation of the p38 MAPK pathway, highlighting its potential therapeutic applications in diabetes management.

## 1. Introduction

As exposure to various radiation scenarios continues to rise, concerns about the potential hazards of ionizing radiation (IR) have intensified. Ionizing radiation has long been acknowledged for its multifaceted effects on biological systems, with outcomes predominantly contingent upon the administered dose. High doses of IR are extensively documented for their cytotoxic effects, resulting in cellular damage and apoptosis [1]. Conversely, low-dose ionizing radiation (LDIR) has garnered interest due to its capacity to elicit adaptive, protective biological responses, a phenomenon known as hormesis [2,3].

According to the VII Report on Biological Effects of Ionizing Radiation (BEIR), doses ranging from 0 to 100 mGy are classified as LDIR [4]. Until recently, it was widely believed that LDIR could precipitate long-term health hazards [5,6]. However, emerging epidemiological, clinical, and preclinical studies have illuminated the potential beneficial health effects of LDIR under both pathological and normal conditions [7,8]. Furthermore, laboratory data suggest that LDIR may confer advantageous effects, including reductions in cancer-related mortality [9,10], enhancements in cellular antioxidant capacity [11], increased longevity across various organisms [12,13], and activation of immune functions [14]. These findings suggest that LDIR is a novel strategy for the clinical prevention and treatment of diseases.

According to the International Diabetes Federation, an estimated 537 million individuals worldwide were living with diabetes in 2021, a number projected to increase to 783 million by 2045 if left unaddressed [15]. Thus, strengthening diabetes prevention and management strategies is of paramount importance. Diabetes mellitus is characterized by chronic metabolic dysregulation, manifesting as persistent hyperglycemia due to absolute or relative deficiencies in insulin secretion [16]. Despite advancements in medical science, diabetes remains a leading cause of morbidity and mortality globally, underscoring the urgent need for innovative therapeutic strategies. Recently, LDIR has attracted attention for its potential benefits in managing diabetes and its associated complications.

Clinical case studies have illustrated improvements in diabetic conditions following LDIR exposure. For instance, radon therapy has been reported to alleviate diabetic symptoms and enhance overall health in patients, as evidenced by elevated levels of superoxide dismutase (SOD) and catalase, increased plasma insulin, and decreased lipid peroxidation and cholesterol levels [17]. Furthermore, research conducted by Kojima et al. [18] described two clinical cases in which patients exhibited improvements in myocardial infarction and glycemic control following radon therapy. Preclinical studies further bolster the therapeutic potential of LDIR, with animal models demonstrating that LDIR can inhibit diabetes onset [19], improve glucose clearance [20], and enhance pancreatic function through upregulation of antioxidant enzymes [21]. Nevertheless, the direct impact of LDIR on pancreatic β-cells and the underlying mechanisms remain to be elucidated.

Given the compelling evidence from both clinical and preclinical investigations, LDIR has emerged as a promising intervention for the management of diabetes. However, comprehensive clinical trials are essential to validate these findings and establish LDIR as a viable therapeutic option. This study aimed to experimentally explore the effects of LDIR on β-cells and investigate the associated mechanisms. The outcomes of this research will provide a foundational basis for the utilization of LDIR as a potential method for the prevention and treatment of diabetes.

## 2. Materials and Methods

### 2.1. Cell Culture and Drug Treatment

The rat RIN-m5F cell line was procured from the Cell Bank of the Chinese Academy of Sciences (Beijing, China). Cells were cultured in RPMI 1640 medium (Thermo Fisher Scientific, Waltham, MA, USA) supplemented with 10% fetal bovine serum (FBS; Shuangru Biotech, Suzhou, China) and 1% penicillin–streptomycin (Solarbio, Beijing, China). All cell cultures were maintained in a 5% CO_2_ incubator (NuAire, Plymouth, MN, USA) at 37 °C with a constant humidity level. For experimental treatments, cells were exposed to glutathione (GSH, 4 mg/mL; Yuanye Biotech, Shanghai, China), hydrogen peroxide (H_2_O_2_, 100 mM), or SB202190 (p38 MAPK inhibitor, 10 µM; Selleckchem, Shanghai, China).

### 2.2. Ionizing Radiation

Cells were subjected to ionizing radiation using an X-ray generator (X-RAD 320 ix, Precision X-ray Inc., North Branford, CT, USA). LDIR levels of 25, 50, and 100 mGy were administered, with a dose rate of 20 mGy/min. High-dose ionizing radiation (HDIR) was set at 2.5 Gy and delivered at a dose rate of 1 Gy/min.

### 2.3. RNA Extraction and Reverse Transcription Quantitative PCR (RT-qPCR)

RNA extraction from RIN-m5F cells and subsequent RT-qPCR were carried out according to established protocols. Total RNA was isolated from cultured cells using Trizol reagent (Beyotime, Shanghai, China) following the manufacturer’s guidelines. One microgram of RNA was reverse transcribed into complementary DNA (cDNA) utilizing the Transcriptor First-Strand cDNA Synthesis Kit (Roche, Indianapolis, IN, USA). Quantitative PCR was performed using SYBR-Green Supermix (Roche, Indianapolis, IN, USA) on an ABI StepOne Plus real-time PCR detection system (Applied Biosystems, Foster City, CA, USA). The relative mRNA expression levels of insulin1 (*Ins1*), insulin2 (*Ins2*), BCL2-associated X protein (*Bax*), Bcl2-like 1 (*Bcl-xl*), and proliferating cell nuclear antigen (*Pcna*) were quantified using the 2^−ΔΔCT^ method [22], normalized to *β-actin* as the reference gene, and compared to the 0 Gy control group. Primers for these genes were custom synthesized by Sangon Biotech (Shanghai, China), with the complete list available in Table 1.

### 2.4. Enzyme-Linked Immunosorbent Assay (ELISA)

Insulin levels in the cultured β-cell supernatants were quantified using the Rat Insulin ELISA Kit (Elabscience, Wuhan, China). Samples and insulin standards were equilibrated to room temperature and added in duplicate to a 96-well ELISA plate. The plate was incubated and washed according to the manufacturer’s instructions. An enzyme-linked secondary antibody was then added, followed by incubation and washing. A substrate solution was added to develop the color reaction. After sufficient color development, the reaction was terminated with a stop solution. The absorbance was measured at 450 nm using a plate reader (Liuyi, Beijing, China). Insulin concentrations in the samples were determined by comparing their absorbance values to a standard curve generated from known insulin concentrations.

### 2.5. Apoptosis Assay

Apoptosis was assessed utilizing the Annexin V-FITC Apoptosis Detection Kit (Beyotime) according to the manufacturer’s instructions. Briefly, cells were irradiated and cultured for 6 h at 37 °C before being harvested. After washing twice with cold phosphate-buffered saline (PBS), the cells were resuspended in 195 μL of binding buffer. Subsequently, 5 μL of Annexin V-FITC was added, and the cells were incubated at room temperature for 15 min. Following incubation, 10 μL of propidium iodide was added, and the cells were incubated further for 10 min. The cells were immediately analyzed using a FACSCanto II flow cytometer (BD Bioscience, Mountain View, CA, USA).

### 2.6. Immunofluorescence

Cells were initially fixed in 4% paraformaldehyde (PFA) for 15 min, followed by a 15 min permeabilization with 0.5% Triton X-100. Subsequent immunofluorescence staining for γH2A.X and phospho-ATM was performed (details are provided in Table 2). The samples were incubated overnight with primary antibodies at 4 °C. After washing, a secondary antibody (Thermo Fisher, Waltham, MA, USA) was applied for 1 h. For negative controls, cells were treated with PBS instead of the primary antibody. Antibody detection and imaging were conducted using a Leica DM6000B fluorescence microscope equipped with Leica LAS X (version 3.7.1) software.

### 2.7. Intracellular Protein Detection by Flow Cytometry

Phospho-ATM detection: Following a 10 min incubation at 37 °C post-ionizing radiation, cells were fixed in 4% PFA for 15 min and then permeabilized with 0.5% Triton X-100 for an additional 15 min. Samples were incubated with a mouse anti-phospho-ATM antibody (details in Table 2) at room temperature for 1 h. After washing, an Alexa Fluor 594-conjugated secondary antibody was applied for 1 h. The cells were subsequently washed and resuspended in PBS for analysis.

Phospho-p38 MAPK detection: Following 2 h of incubation at 37 °C post-ionizing radiation, cells underwent the same fixation and permeabilization process. They were then incubated with a phospho-p38 MAPK (Thr180, Tyr182) rabbit monoclonal antibody, PE (details in Table 2), at room temperature for 1 h. After washing, cells were resuspended in PBS for evaluation.

Flow cytometry: Flow cytometric analyses were conducted using a BD-FACSCanto II flow cytometer, and the results were analyzed using FlowJo X (version 10.07) software (TreeStar, Ashland, OR, USA).

### 2.8. 5-Ethynyl-2′-Deoxyuridine (EdU) Incorporation Assay

DNA synthesis was evaluated using the BeyoClick™ EdU Cell Proliferation Kit (Beyotime) according to the manufacturer’s instructions. Cultured cells were seeded into 6-well plates, and 2 h after exposure to ionizing radiation, they were incubated with 10 μM EdU solution at 37 °C for 2 h. Following incubation, cells were harvested and fixed with 4% PFA for 15 min. Azide 594 working solution was subsequently added to induce a fluorescent color reaction. Fluorescence detection was performed using a FACSCanto II flow cytometer.

### 2.9. CCK-8 Assay

Cells were seeded in a 96-well plate at a density of 2000 cells per well in 100 μL of RPMI 1640 medium. After adherence, the cells were subjected to ionizing radiation treatment. At 24, 48, 72, and 96 h post-irradiation, 10 μL of CCK-8 reagent (Beyotime) was added to each well, followed by a 2 h incubation at 37 °C. Optical density at 450 nm was subsequently measured using a microplate reader (Liuyi, Beijing, China).

### 2.10. Reactive Oxygen Species (ROS) Detection

ROS levels were assessed at 0 h and 3 h post-ionizing radiation exposure. ROS detection in adherent cells was accomplished using the oxidative fluorescent dye dihydroethidium (DHE; Beyotime, Shanghai, China). Following a PBS wash, cells were stained with 5 μM DHE at 37 °C in the dark for 30 min, as per the manufacturer’s instructions. ROS generation was analyzed using a Leica DM6000B fluorescence microscope equipped with Leica LAS X software, and quantitative analysis was performed using ImageJ (version 1.60) [23].

For flow cytometric analysis, cultured cells were harvested from 6-well plates after various ionizing radiation treatments. After the culture medium was removed, cells were incubated in serum-free RPMI 1640 medium containing 10 μM DCFH-DA at 37 °C for 20 min. Subsequently, the cells were washed three times with serum-free medium to eliminate excess DCFH-DA and immediately analyzed using a FACSCanto II flow cytometer.

### 2.11. Western Blotting

RIN-m5F cells were irradiated and subsequently cultured at 37 °C for either 0.5 h (for γH2A.X detection) or 4 h (for insulin and PDX-1 detection) before being harvested. Total protein was extracted using RIPA lysis buffer (Beyotime), and protein concentrations were determined using an enhanced BCA protein assay kit (Beyotime). Equal amounts of protein (20 μg) were separated by SDS-PAGE and transferred to polyvinylidene fluoride (PVDF) membranes. The membranes were blocked with QuickBlock™ Blocking Buffer (Beyotime) for 15 min and then incubated with primary antibodies against γH2A.X, insulin, PDX-1, and β-tubulin (Table 2). HRP-conjugated goat anti-mouse IgG or anti-rabbit IgG (Huabio, Hangzhou, China) served as the secondary antibody. Protein band intensities were quantified using ImageJ software (version 1.60) [24].

### 2.12. Statistical Analysis

Treatment and data analyses were conducted in a blinded manner with masked sample labels. Statistical analyses were performed using GraphPad Prism 7.0 (GraphPad, San Diego, CA, USA) software. All experiments were repeated 3 or 4 times independently, with the data presented as the mean of biological replicates. Data were expressed as mean ± standard deviation (SD). Homogeneity of variance was evaluated using the F-test. Differences among three or more groups were analyzed using one-way analysis of variance (ANOVA). Statistical significance was defined as a *p*-value of less than 0.05.

## 3. Results

### 3.1. A Single Exposure to Ionizing Radiation Leads to an Increase in Insulin Synthesis by β-Cells Within a Short Period

**Figure 1:**
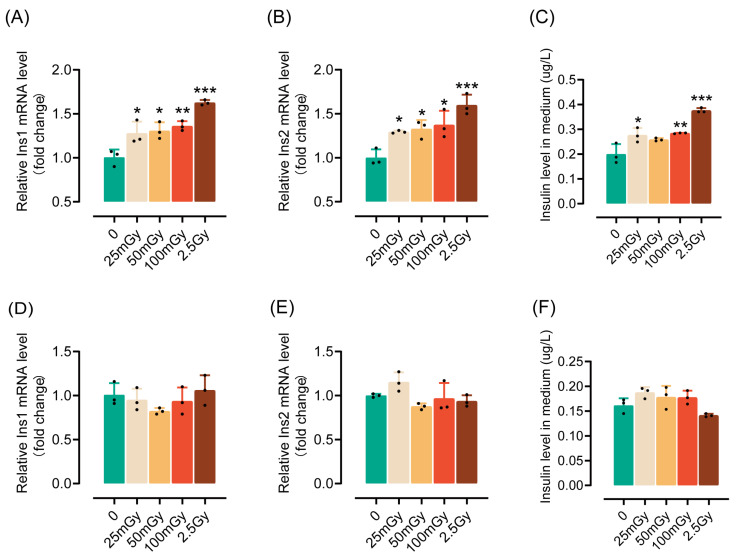
A single exposure to ionizing radiation leads to an increase in insulin synthesis by β-cells within a short period. β-cells were irradiated and cultured for either 2 or 6 h (h) at 37 °C, after which insulin levels were measured. The mRNA levels of *Ins1* (**A**), *Ins2* (**B**), and insulin content in the medium (**C**) measured 2 h post-irradiation. (**D**–**F**) The mRNA levels of *Ins1* (**D**), *Ins2* (**E**), and insulin content in the medium (**F**) measured 6 h post-irradiation (4 h after ionizing radiation, the medium was replaced with fresh medium; after an additional 2 h of incubation, the supernatant was collected to measure insulin levels). Results are shown as mean ± SD (*n* = 3). * *p* < 0.05, ** *p* < 0.01, and *** *p* < 0.001 vs. the 0 Gy group.

To investigate the impact of ionizing radiation on the insulin synthesis and secretion functions of pancreatic β-cells, rat RIN-m5F cells were exposed to various levels of low-dose and high-dose ionizing radiation. Compared to non-irradiated cells, both low and high doses of ionizing radiation significantly increased insulin mRNA expression two hours post-irradiation (Figure 1A,B). Additionally, the insulin content in the medium was notably elevated (Figure 1C), indicating that ionizing radiation not only enhances insulin expression but also promotes its secretion. However, by six hours post-irradiation, the ability of the cells to express and secrete insulin returned to levels observed in non-irradiated cells (Figure 1D–F). In summary, these results suggest that a single exposure to ionizing radiation can transiently activate the insulin synthesis and secretion capabilities of pancreatic β-cells. Given that a dose of 25 mGy significantly enhances the insulin synthesis function of β-cells, we utilized 25 mGy as the LDIR group in subsequent experiments.

### 3.2. LDIR Do Not Induce Apoptosis in RIN-m5F Cells

**Figure 2:**
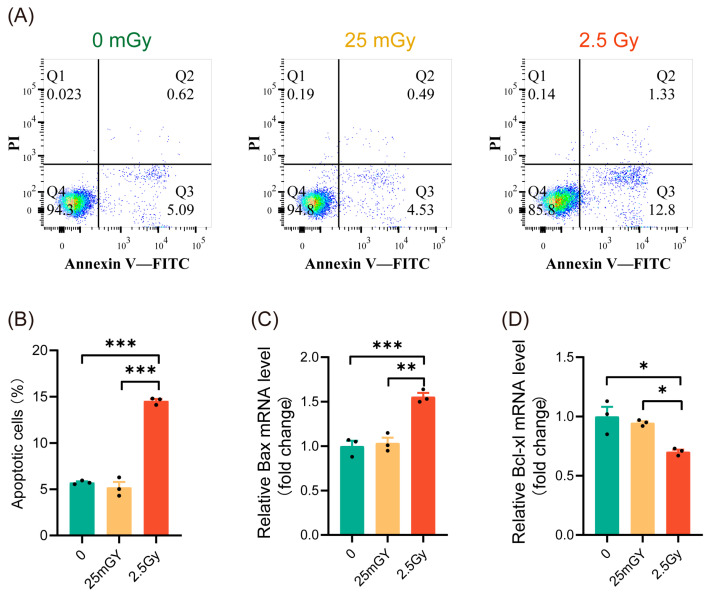
LDIR does not induce apoptosis in RIN-m5F cells. Cell apoptosis and the expression of apoptosis-related genes detected 6 h post-irradiation. (**A**) Representative flow cytometry data. (**B**) Statistical analysis of cell apoptosis rates. The mRNA levels of *Bax* (**C**) and *Bcl-xl* (**D**). Results are shown as mean ± SD (*n* = 3). * *p* < 0.05, ** *p* < 0.01, and *** *p* < 0.001.

Previous studies have indicated that ionizing radiation can influence cell apoptosis [25]. To characterize the mechanisms activated by LDIR in β-cells, we investigated whether LDIR induces apoptosis. Apoptosis analysis showed that 2.5 Gy irradiation significantly increased apoptosis in RIN-m5F cells (Figure 2A,B, red histograms). In contrast, compared to non-irradiated β-cells, 25 mGy LDIR did not significantly affect the apoptosis rate (Figure 2A,B, yellow histograms). To further confirm apoptosis levels, we examined the expression of the pro-apoptotic gene *Bax* and the anti-apoptotic gene *Bcl-xl*. The results indicated that high-dose ionizing radiation promoted Bax expression (Figure 2C, red histograms) while decreasing the expression of *Bcl-xl* (Figure 2D, red histograms). LDIR, however, did not significantly alter the expression levels of these genes (Figure 2C,D, yellow histograms). These findings suggest that a single exposure to LDIR does not induce apoptosis in cells.

### 3.3. LDIR Does Not Modify Cell Proliferation in RIN-m5F Cells

**Figure 3:**
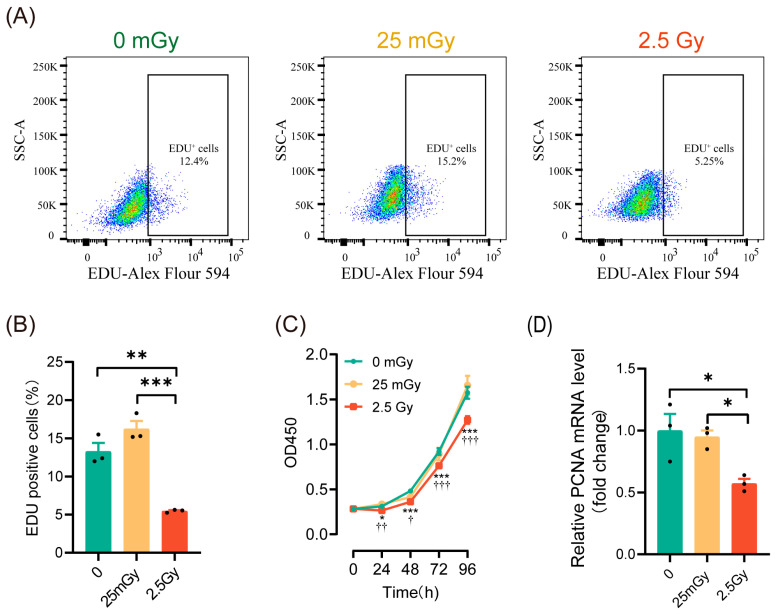
LDIR does not modify cell proliferation in RIN-m5F cells. Two hours post-irradiation, cells were labeled with EdU to assess DNA replication and to evaluate cell proliferation, as well as proliferation-related genes. (**A**) Representative flow cytometry data. (**B**) Statistical analysis of cells undergoing DNA replication. ** *p* < 0.01 and *** *p* < 0.001. (**C**) CCK-8 assay for cell proliferation. * *p* < 0.05, and *** *p* < 0.001, 0 Gy vs. 2.5 Gy; † *p* < 0.05, †† *p* < 0.01, and ††† *p* < 0.001, 25 mGy vs. 2.5 Gy. (**D**) The mRNA level of *Pcna*. * *p* < 0.05. All data are shown as mean ± SD (*n* = 3).

To investigate the effects of ionizing radiation on β-cell proliferation, we initially employed EdU labeling to detect the proportion of cells undergoing DNA replication within the cell cycle. The results (Figure 3A,B) indicated that DNA replication was significantly inhibited in cells treated with 2.5 Gy irradiation. In contrast, the percentage of EdU-labeled cells in the 25 mGy LDIR group did not decrease and even exhibited a slight increasing trend (*p* > 0.05). We then assessed β-cell viability using the CCK-8 assay, which revealed a significant decrease in cell viability in the 2.5 Gy irradiation group, while no significant change was observed in the 25 mGy LDIR group (Figure 3C). To further validate these findings, we examined the expression of the proliferation-related gene *Pcna* (proliferating cell nuclear antigen). The results demonstrated that *Pcna* expression significantly decreased in the high-dose irradiation group, whereas no significant change was noted in the low-dose irradiation group (Figure 3D). Collectively, these data suggest that high-dose ionizing radiation significantly inhibited β-cell proliferation, whereas low-dose ionizing radiation did not exert a noticeable impact on proliferation.

### 3.4. LDIR Does Not Induce DNA Double-Strand Breaks nor Activate DNA Repair Pathways in RIN-m5F Cells

**Figure 4:**
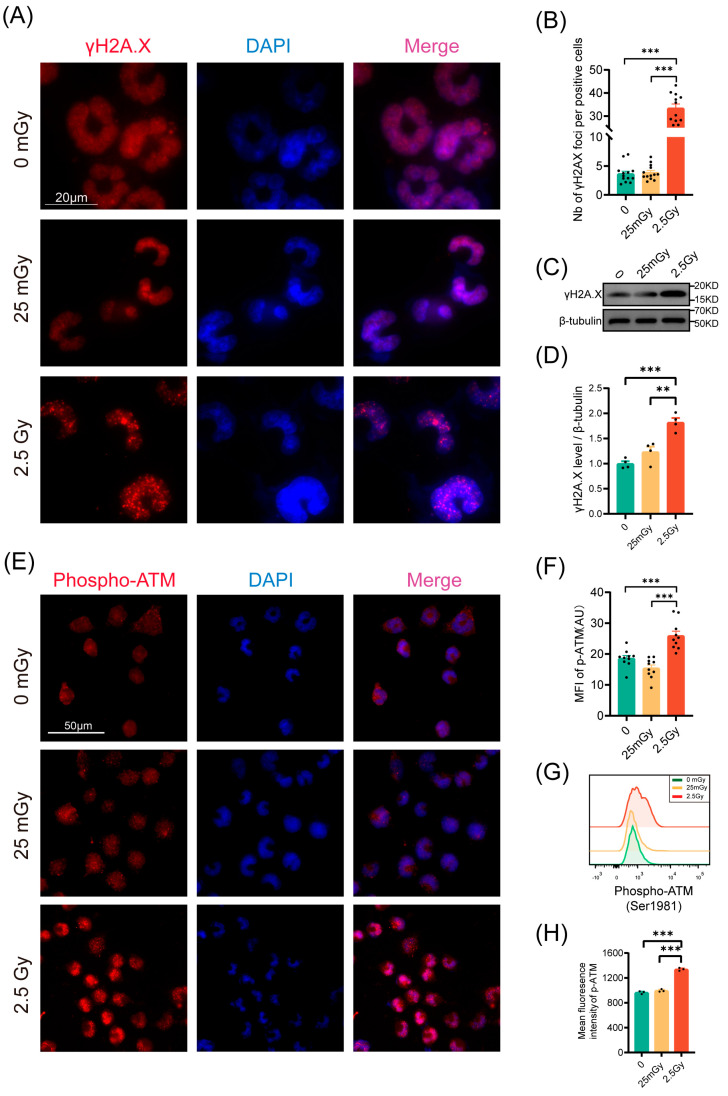
LDIR does not induce DNA double-strand breaks nor activate the DNA repair pathway in RIN-m5F cells. (**A**) γH2A.X foci were examined by immunofluorescence 30 min post-IR. For each condition, all cells in at least 10 fields of view were analyzed. Blue: DAPI; Red: γH2A.X. (**B**) Number of γH2A.X foci by positive cells. Western blot (WB) analysis of γH2A.X protein levels, including representative bands (**C**) and quantitative data (**D**), *n* = 4. RIN-m5F cells were irradiated and cultured for 10 min at 37 °C. (**E**–**H**) Analysis of ATM-phosphorylation on Ser1981 by immunofluorescence and flow cytometry in RIN-m5F cells. Phospho-ATM immunofluorescence representative images (**E**) and quantitative data (**F**), *n* = 10 fields. Blue: DAPI; Red: Phospho-ATM. Representative flow cytometry data (**G**) and statistical analysis of ATM phosphorylation (**H**), *n* = 3. ** *p* < 0.01 and *** *p* < 0.001.

Given that irradiation typically induces DNA double-strand breaks, we quantified the number of γH2A.X foci 30 min post-irradiation via immunofluorescence (Figure 4A). Compared to 2.5 Gy irradiation, exposure to 25 mGy did not increase the number of γH2A.X foci relative to unirradiated β-cells, indicating that 25 mGy LDIR does not cause DNA double-strand breaks (Figure 4B). Consistently, Western blot results also showed that the levels of phosphorylated γH2A.X protein did not change significantly in the LDIR group, while a substantial increase was observed in the HDIR group (Figure 4C,D). We further investigated the DNA damage response pathway post-LDIR exposure by measuring ATM phosphorylation 10 min after irradiation via immunofluorescence and flow cytometry. As anticipated, cells exposed to 2.5 Gy displayed a significant increase in ATM phosphorylation compared to control cells (Figure 4E–H). Conversely, no increase in ATM phosphorylation was observed following exposure to 25 mGy (Figure 4E–H). These findings collectively suggested that 25 mGy LDIR neither induced DNA double-strand breaks nor activated the ATM-dependent DNA damage repair pathway in RIN-m5F cells.

### 3.5. LDIR Induces a Transient Increase in Reactive Oxygen Species (ROS)

**Figure 5:**
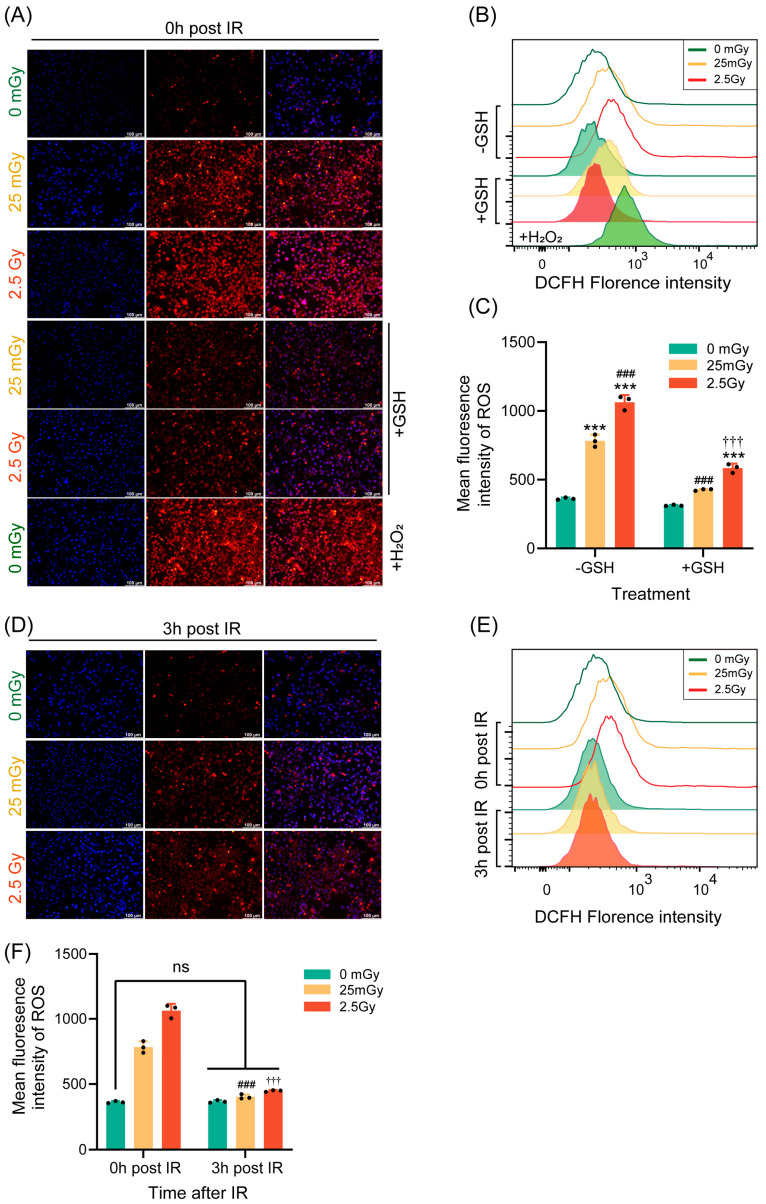
LDIR induces a transient increase in ROS. ROS levels were quantified in RIN-m5F cells immediately (**A**–**C**) or 3 h (**D**–**F**) after IR. (**A**,**D**) Detection of ROS in adherent cells using dihydroethidium (DHE), with H_2_O_2_ as a positive intervention for ROS and GSH as an antioxidant to inhibit ROS production. Blue: DAPI; Red: DHE. Flow cytometric detection of ROS was performed using the fluorescent probe 2**′**,7**′**-dichlorodihydrofluorescein diacetate (DCFH-DA). Overlay histograms (**B**,**E**) represent DCFH fluorescence. (**C**,**F**) Mean fluorescence intensity of 2**′**,7**′**-dichlorodihydrofluorescein (DCFH). Results are presented as mean ± SD, *n* = 3. *** *p* < 0.001 vs. 0 Gy without GSH; ^###^ *p* < 0.001 vs. 25 mGy without GSH; ^†††^ *p* < 0.001 vs. 2.5 Gy without GSH; ns *p* > 0.05.

Ionizing radiation has been widely shown to promote ROS production within cells, with moderate oxidative stress capable of activating various cellular physiological functions [26]. Therefore, we investigated the impact of LDIR on ROS production in β-cells. Immediately following ionizing radiation, ROS production was measured using DHE fluorescence staining and flow cytometry. Compared with those in non-irradiated cells, ROS levels were significantly elevated in cells exposed to both low and high doses of radiation. Notably, ROS production was higher in cells exposed to the high dose of 2.5 Gy compared to those exposed to the low dose of 25 mGy (Figure 5A–C). Additionally, the ROS induced by both low-dose and high-dose ionizing radiation were significantly inhibited by the antioxidant GSH (Figure 5A–C).

Given that the effect of ionizing radiation on insulin expression was transient, we also examined whether its effect on ROS levels was similarly transient. Thus, we measured ROS production 3 h post-irradiation using the same methodologies. The results demonstrated that ROS levels in both the LDIR and HDIR groups returned to baseline levels, equivalent to those in non-irradiated cells, after 3 h (Figure 5D–F). In summary, a single exposure to ionizing radiation transiently increased ROS levels in β-cells.

### 3.6. Ionizing Radiation Promotes β-Cell Function Through the ROS/p38 MAPK Pathway

**Figure 6:**
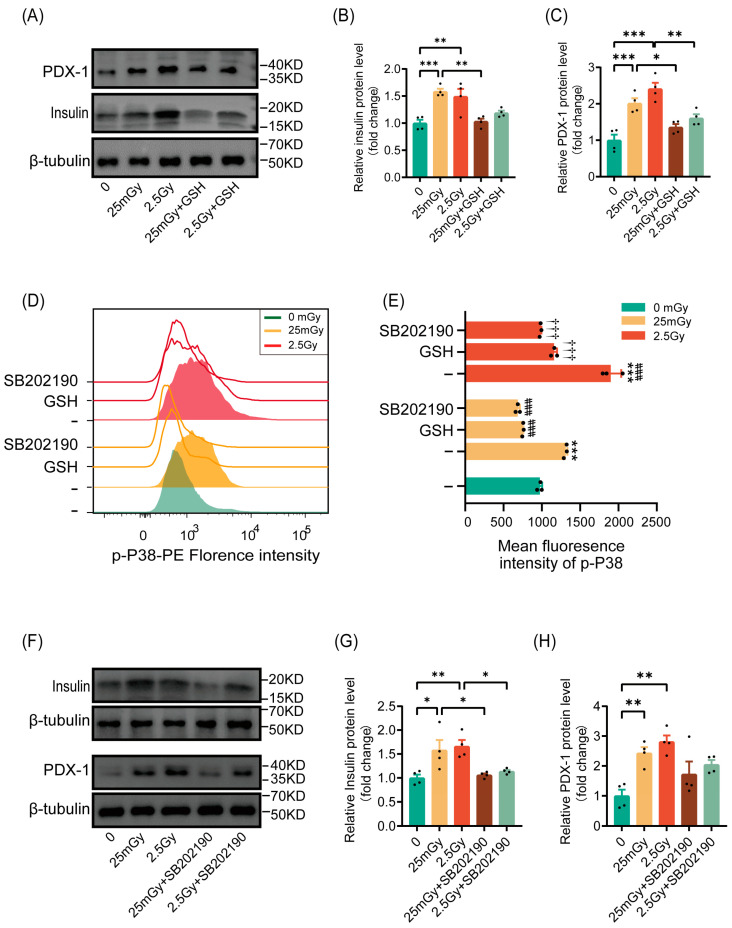
Ionizing radiation promotes β-cell function through the ROS/p38 MAPK pathway. Cells were irradiated and cultured for 2 h at 37 °C. (**A**–**C**) Western blot data of PDX-1 and insulin (results are normalized to those in 0 Gy conditions, *n* = 4). Cells were pre-treated or not with GSH prior to IR. * *p* < 0.05, ** *p* < 0.01, and *** *p* < 0.001. Phosphorylation of p38 MAPK on Thr180/Tyr182 was analyzed by flow cytometry. Overlay histograms (**D**) represent one representative experiment out of three. Histogram bars (**E**) show the mean fluorescence intensity (MFI) of phospho-p38 MAPK in RIN-m5F cells (*n* = 3). *** *p* < 0.001 vs. 0 Gy without GSH; ^###^ *p* < 0.001 vs. 25 mGy without GSH; ^†††^ *p* < 0.001 vs. 2.5 Gy without GSH. Cells were pre-treated with GSH or SB202190, or left untreated, prior to IR. (**F**–**H**) Western blot data of PDX-1 and insulin (results are normalized to those in 0 Gy conditions, *n* = 4). Cells were pre-treated or not with SB202190 prior to IR. * *p* < 0.05 and ** *p* < 0.01. All data are shown as mean ± SD.

Based on our findings that ionizing radiation transiently elevated ROS levels and activated β-cell function, we investigated whether radiation would activate insulin synthesis in β-cells through ROS. The results indicated that pre-treatment with the antioxidant GSH inhibited the radiation-induced increases in insulin and the expression of the insulin-synthesis-related gene PDX-1 (Figure 6A–C). These findings confirmed that ionizing radiation enhanced insulin synthesis in β-cells via ROS activation.

We then explored whether ROS leads to the activation of p38 MAPK, as previously reported. Phospho-p38 MAPK, a marker of p38 MAPK pathway activation, was significantly increased in cells after ionizing radiation at both low and high doses, with higher levels observed under high-dose conditions compared to the 25 mGy LDIR group. However, cells pre-treated with antioxidants exhibited no significant increase in p38 MAPK phosphorylation following radiation, paralleling the results obtained with the p38 MAPK pathway inhibitor SB202190 (Figure 6D,E). This suggests that ionizing radiation activated the p38 MAPK pathway through ROS.

Previous reports have indicated that the p38 MAPK pathway regulates insulin synthesis by controlling PDX-1 levels. We verified this by inhibiting the p38 MAPK pathway with SB202190 prior to irradiation. The results showed that ionizing radiation did not significantly increase the expression of insulin or the insulin-transcription-related protein PDX-1 (Figure 6F–H). In summary, ionizing radiation promoted β-cell function through the activation of the ROS/p38 MAPK pathway.

### 3.7. HDIR Induces Apoptosis Through Overactivation of the ROS/p38 MAPK Pathway

**Figure 7:**
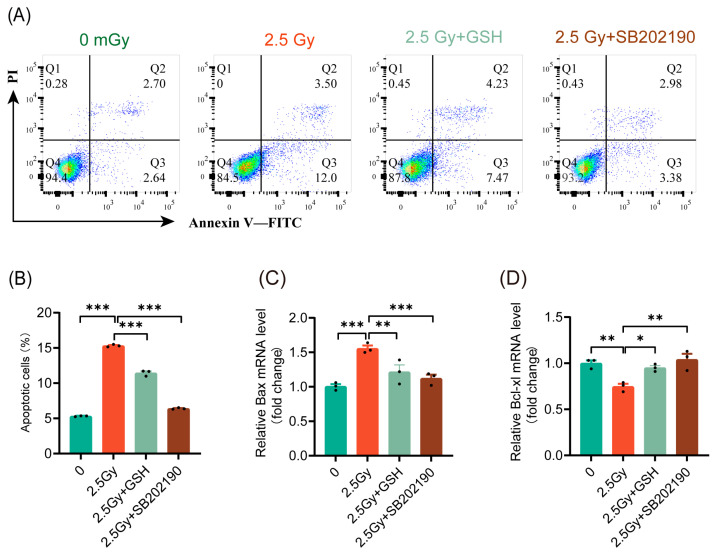
HDIR induces apoptosis through overactivation of the ROS/p38 MAPK pathway. Cell apoptosis and the expression of apoptosis-related genes detected 6 h post-irradiation. (**A**) Representative flow cytometry data. (**B**) Statistical analysis of cell apoptosis rates. The mRNA levels of *Bax* (**C**) and *Bcl-xl* (**D**). Results are shown as mean ± SD (*n* = 3). * *p* < 0.05, ** *p* < 0.01, and *** *p* < 0.001.

Various studies have demonstrated inconsistent roles of the p38 MAPK pathway in cell apoptosis [27]. Our results indicated that ROS induced by different doses of ionizing radiation activated the p38 MAPK pathway to varying extents. Under LDIR, apoptosis did not significantly increase, whereas HDIR resulted in a notable increase in cell apoptosis. These findings prompted us to investigate whether HDIR induces apoptosis through ROS-mediated activation of the p38 MAPK pathway. The results indicated that both antioxidant treatment and p38 MAPK pathway inhibition significantly reduced the rate of apoptosis induced by 2.5 Gy HDIR (Figure 7A,B). Similarly, both antioxidant treatment and p38 MAPK pathway inhibition suppressed the expression of the pro-apoptotic gene *Bax* (Figure 7C) and promoted the expression of the anti-apoptotic gene *Bcl-xl* (Figure 7D). These findings indicated that HDIR induced apoptosis via ROS activation of the p38 MAPK pathway.

## 4. Discussion

In this study, we observed that a single exposure to both 25 mGy of LDIR and 2.5 Gy of HDIR transiently enhanced insulin synthesis and secretion in pancreatic β-cells, with effects returning to baseline levels by six hours post-exposure. While LDIR did not induce apoptosis or alter cell proliferation, HDIR significantly promoted apoptosis and inhibited proliferation. Additionally, LDIR did not cause DNA double-strand breaks or activate the DNA repair pathway, in contrast with the effects observed with HDIR. Our findings also revealed that ionizing radiation transiently elevated ROS levels, which in turn activated the p38 MAPK pathway to increase insulin synthesis. However, HDIR induced apoptosis through overactivation of the ROS/p38 MAPK pathway. These results underscore the dose-dependent effects of ionizing radiation on β-cell function and viability, highlighting the complex roles of ROS and p38 MAPK signaling in mediating these responses.

Ionizing radiation has been demonstrated to exert protective effects on diabetes. A study by Takehara et al. [19] confirmed that LDIR has anti-diabetic effects in diabetic mice, reporting that LDIR pre-treatment inhibited thymidine-induced diabetes by increasing antioxidant levels and protecting pancreatic cells, thereby delaying the onset of hyperglycemia. Ionizing radiation has been found to improve insulin sensitivity and lower blood glucose levels in diabetic mice, potentially due to radiation-induced cellular repair mechanisms [28]. Furthermore, ionizing radiation may alleviate diabetes-related metabolic disorders by affecting adipose tissue function [28]. However, the dose and duration of ionizing radiation are critical for determining its effects. LDIR may confer protective benefits, whereas HDIR can induce cell damage and exacerbate diabetes [29]. Our study revealed that a single exposure to ionizing radiation can temporarily increase insulin synthesis and secretion in pancreatic β-cells. However, this effect is transient, with expression and secretion capacity returning to baseline levels after six hours. This phenomenon suggests that ionizing radiation may promote insulin synthesis and secretion by transiently activating specific molecular signaling pathways. A deeper understanding of the mechanisms behind this transient response could lead to new strategies aimed at improving β-cell function in diabetic patients, leveraging the potential protective effects of ionizing radiation.

As a potential new strategy for diabetes prevention and treatment, the dose of ionizing radiation should not cause damage to β-cells. We examined the effects of two doses of ionizing radiation on apoptosis and cell proliferation. The results indicated that 25 mGy of LDIR did not significantly increase the apoptosis rate nor alter the expression levels of the pro-apoptotic gene *Bax* and the anti-apoptotic gene *Bcl-xl*. These findings indicated that LDIR can modulate pancreatic β-cell functions without inducing apoptosis. It is well established that HDIR can induce apoptosis and inhibit cell proliferation [25,30]. However, the effects of low-dose ionizing radiation on apoptosis and proliferation remain controversial, potentially due to species-specific and cell-specific sensitivities to LDIR. For instance, exposure to 10–100 mGy of X-rays in embryonic mouse brains significantly increased apoptosis in embryonic neural stem cells [31]. Conversely, a study on human hematopoietic stem cells demonstrated that 20 mGy of LDIR did not increase apoptosis or affect the cell cycle [32], consistent with our findings. Specifically, 25 mGy of LDIR did not significantly affect DNA replication or cell viability, while HDIR (2.5 Gy) significantly inhibited cell proliferation, as further confirmed by analysis of *Pcna* expression. Other studies have shown that, under certain conditions, LDIR is even considered to have a “stimulatory” effect, capable of promoting cell proliferation and function. For example, LDIR can induce adaptive responses in cells, enhancing their resistance to subsequent HDIR [33]. Radiation adaptive response refers to a phenomenon where exposure to low-dose radiation reduces the sensitivity of organisms or cells to subsequent higher doses of radiation. This suggests that low-dose radiation can activate protective mechanisms, such as antioxidant defenses, helping the organism better cope with radiation-induced damage [3]. Furthermore, LDIR may help maintain the cell proliferation capacity by regulating the cell cycle and promoting the expression of anti-apoptotic factors [34].

In addition to affecting apoptosis and cell proliferation, DNA damage can impair normal cellular functions. Our analysis of classical DNA damage markers revealed that 25 mGy of LDIR did not cause DNA double-strand breaks or activate the ATM-dependent DNA damage repair pathway. In contrast, HDIR significantly increased the expression of these DNA damage markers. Although ionizing radiation is widely recognized as a primary cause of DNA damage, studies indicated that the cellular response to low doses may not be as significant as expected. Henry et al. [32] found that 20 mGy of LDIR did not induce the classical DNA damage and repair pathways typically activated by γ-irradiation. LDIR may trigger adaptive cellular responses rather than directly causing DNA double-strand breaks [35]. For instance, certain cells exhibited hyper-radiosensitivity (HRS) after low-dose exposure but demonstrate radio-resistance at slightly higher doses [36]. Moreover, LDIR might mediate bystander and adaptive responses by inducing the release of oxidized cell-free DNA (cfDNA), suggesting that its biological effects may not be directly related to DNA double-strand breaks [37]. Thus, while high-dose ionizing radiation leads to DNA double-strand breaks, the mechanisms of cellular response at low doses are more complex, potentially affecting cell survival and repair through alternative biological pathways [38].

Radiolysis of intracellular water and subsequent oxidative stress caused by the generation of highly reactive free radicals are the primary reasons for cellular damage in response to radiation [39]. HDIR typically leads to direct DNA damage and cell death, primarily through the generation of substantial amounts of ROS that rapidly form following radiation exposure, causing acute cellular injury [26]. In contrast, the effects of low-dose ionizing radiation are more intricate. While it also generates ROS, the mechanisms of ROS production and the biological effects differ from those of HDIR. LDIR may influence cellular function by inducing adaptive responses and long-term metabolic changes, and in certain cases, it may even exhibit beneficial biological effects [8,40]. Our study found that LDIR induced a transient increase in ROS in pancreatic β-cells. This is consistent with the established role of ionizing radiation in promoting intracellular ROS generation, as observed in previous studies [32]. Importantly, both low-dose (25 mGy) and high-dose (2.5 Gy) radiation resulted in significantly elevated ROS levels compared to non-irradiated cells, with a more pronounced increase in the high-dose group. Notably, this effect was transient, with ROS levels returning to baseline within three hours after radiation exposure, which corresponds with the transient enhancement of insulin synthesis and secretion that we observed. These findings suggested that the initial increase in ROS may serve as a signaling mechanism that temporarily enhances β-cell function, while sustained or excessive ROS generation induced by HDIR could lead to cellular damage and apoptosis. This highlights the dual role of ROS in cellular physiology and the stress response, emphasizing the importance of a nuanced understanding of oxidative stress within the context of radiation exposure.

It has been widely demonstrated that ionizing radiation affects various physiological functions of cells through oxidative stress [41,42]. Although ROS are generally considered harmful to β-cells, there is evidence suggesting their potential positive or necessary role in normal β-cell function. A study by Penicaud and colleagues demonstrated that mitochondria-derived ROS are essential for normal glucose-stimulated insulin secretion. In that study, antioxidants inhibited insulin secretion; however, insulin release could still be induced via a mitochondrial complex inhibitor that generates ROS [43]. Another study confirmed these findings, revealing that ROS-induced calcium ion release is a crucial step in glucose-induced insulin secretion [44]. Our data also revealed that ionizing radiation transiently enhanced insulin expression and, similarly, the antioxidant GSH suppressed insulin expression, indicating that ionizing radiation may exert its effects by increasing ROS activity.

Oxidative stress triggers cellular responses by activating multiple protein phosphorylation pathways, including the MAPK pathway, specifically stress-activated protein kinases (SAPKs), with p38 MAPK being one of them. We found that ionizing radiation significantly increased p38 MAPK phosphorylation levels, and this effect was suppressed by the antioxidant GSH and the p38 MAPK inhibitor SB202190. These findings suggested that ionizing radiation activated the p38 MAPK pathway through ROS. Additionally, we discovered that activation of the p38 MAPK pathway promoted the expression of the insulin transcription factor PDX-1, thereby increasing insulin levels. Research on the impact of the p38 MAPK pathway on insulin expression is inconsistent. The majority of studies indicate that the p38 MAPK pathway inhibits β-cell insulin production [45,46]. However, other studies support our findings. Song et al. [47] reported that the overexpression of iPLA2β enhances insulin secretion by activating p38 MAPK in INS-1 insulinoma cells and isolated islets, and pharmacological inhibition of p38 MAPK prevents this effect. In human islets or MIN6 β-cells, specific inhibition of stress-activated p38 MAPK by SB103580 inhibits the binding of PDX-1 to DNA and PDX-1-dependent gene transcription in response to glucose stimulation [48,49]. Additionally, overexpression of p38 kinase mimics the impact of glucose on PDX-1 binding and stimulates transcription of the human insulin promoter. Another study demonstrated that p38 MAPK serves as a positive regulator of PDX-1 stability, exerting its effect by inhibiting PDX-1 ubiquitination in a phosphorylation-dependent manner [50]. We speculate that the inconsistencies among different studies may be attributed to variations in the stimulation time and the extent of p38 MAPK activation.

Activation of the p38 MAPK pathway is believed to be a causative factor for apoptosis in pancreatic β-cells [27]. Our study demonstrated that HDIR significantly increased cell apoptosis through the overactivation of the ROS/p38 MAPK pathway. Both antioxidant treatment and p38 MAPK pathway inhibition significantly reduced apoptosis induced by 2.5 Gy HDIR and regulated the expression of *Bax* and *Bcl-xl* genes. Interestingly, while LDIR also activated the p38 MAPK pathway, it did not increase cell apoptosis. This may be attributed to the significantly lower level of p38 MAPK activation induced by LDIR compared to HDIR. It is reasonable to believe that the differential activation levels of p38 MAPK led to distinct outcomes concerning apoptosis.

## 5. Conclusions

**Figure 8:**
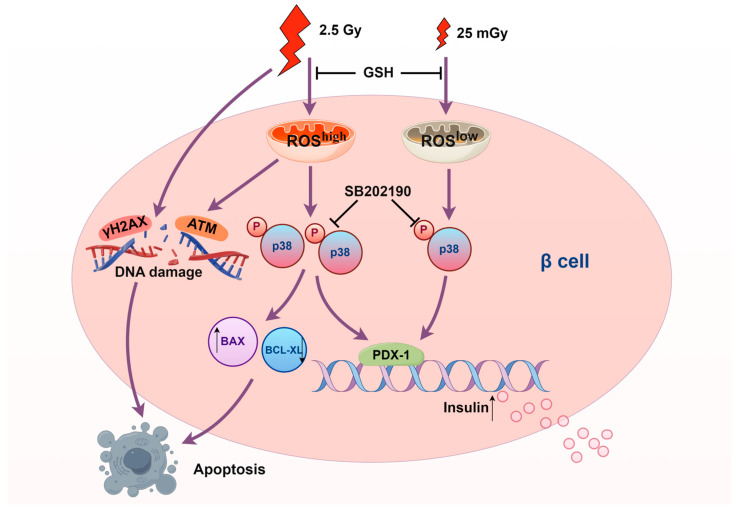
Scheme of the molecular mechanisms underlying the effects of ionizing radiation on pancreatic β-cells (produced by Figdraw 2.0).

Our findings have important implications for understanding the dose-dependent effects of ionizing radiation on pancreatic β-cells (Figure 8). This study may provide new insights into protective mechanisms for beta cells, such as in the context of β-cell transplantation or strategies to safeguard remaining β-cells. For type 2 diabetes, although early stages are characterized by insulin resistance, the later stages involving β-cell dysfunction and exhaustion might benefit from interventions aimed at preserving beta-cell function. This study provided a basis for exploring low-dose radiation as a potential tool to protect beta cells and combat their dysfunction in the progression of type 2 diabetes. However, the risk of HDIR-induced apoptosis underscores the need for caution and precise dose management in any therapeutic application. Future studies should aim to further elucidate the molecular mechanisms underlying the differential activation of the p38 MAPK pathway by varying levels of ROS. Investigating other signaling pathways involved and their interactions with p38 MAPK could provide a more comprehensive understanding of radiation-induced cellular responses. Additionally, in vivo studies are necessary to confirm our in vitro findings and assess the translational potential of LDIR therapy in clinical settings.

In conclusion, our study highlighted the dose-dependent effects of ionizing radiation on β-cell function and apoptosis, which were mediated through the ROS/p38 MAPK pathway. These findings contribute to a deeper understanding of radiation biology and suggest potential applications in type 2 diabetes treatment, while also cautioning against the detrimental effects of HDIR.

## Figures and Tables

**Table 1:** Sequences of the primers used for RT-qPCR.

Gene	Accession Number	Forward Primer (5′–3′)	Reverse Primer (5′–3′)
*Ins1*	NM_019129	ACAACTGGAGCTGGGTGGAGG	GTTGCAGTAGTTCTCCAGTTGGTAGAG
*Ins2*	NM_019130	CAGCACCTTTGTGGTTCTCA	AGAGCAGATGCTGGTGCAG
*Bax*	NM_017059	CTGGACAACAACATGGAG	AAGTAGAAAAGGGCAACC
*Bcl-xl*	NM_001033670	TAGGTGGTCATTCAGGTAGG	GTGGAAAGCGTAGACAAGG
*Pcna*	NM_022381	AAGTTTTCTGCGAGTGGGGA	ACAGTGGAGTGGCTTTTGTGA
*β-actin*	NM_031144	GTCGTACCACTGGCATTGTG	CTCTCAGCTGTGGTGGTGAA

**Table 2:** Antibodies used for immunostaining, Western blotting, and flow cytometry.

Antibody Name	Catalog No.	Vendor	Application and Dilution
Primary antibody			
Rabbit anti-insulin antibody	A19066	ABclonal (Wuhan, China)	1:1000 (WB)
Rabbit anti-pdx-1 antibody	20989-1-AP	Proteintech (Wuhan, China)	1:1000 (WB)
Rabbit anti-phospho-Histone H2A.X (ser139) (γH2A.X) antibody	381558	Zenbio (Chengdu, China)	1:200 (IF), 1:1000 (WB)
Mouse anti-phospho-ATM antibody	AA866	Biotime (Shanghai, China)	1:200 (IF), 1:100 (Flow)
Rabbit anti-phospho-p38 MAPK (Thr180, Tyr182) antibody, PE	MA5-36912	Invitrogen (Waltham, MA, USA)	5 µL/1 × 10^6^ cells (Flow)
Mouse anti-β-tubulin antibody	66240-1-Ig	Proteintech	1:20,000 (WB)
Secondary antibody			
Alexa Fluor 594 goat anti-rabbit IgG (H + L) antibody	A11012	ThermoFisher (Waltham, MA, USA)	1:1000 (IF), 1:500 (Flow)
Alexa Fluor 594 goat anti-mouse IgG (H+ L) antibody	A11005	ThermoFisher	1:1000 (IF), 1:500 (Flow)

IF: immunofluorescence; WB: Western blotting; Flow: flow cytometry.

## Data Availability

Correspondence and requests for materials should be addressed to Zhonglu Liao.
